# A systematic review evaluating the clinimetric properties of the Victorian Institute of Sport Assessment (VISA) questionnaires for lower limb tendinopathy shows moderate to high-quality evidence for sufficient reliability, validity and responsiveness—part II

**DOI:** 10.1007/s00167-021-06557-0

**Published:** 2021-04-16

**Authors:** Vasileios Korakakis, Rod Whiteley, Argyro Kotsifaki, Manos Stefanakis, Yiannis Sotiralis, Kristian Thorborg

**Affiliations:** 1 Aspetar Orthopaedic and Sports Medicine Hospital, 29222 Doha, Qatar; 2 Hellenic Orthopaedic Manipulative Therapy Diploma (HOMTD), Athens, Greece; 3 School of Science, Program of Physiotherapy, University of Nicosia, Nicosia, Cyprus; 4 Department of Orthopaedic Surgery, Sports Orthopedic Research Center-Copenhagen (SORC-C), Amager-Hvidovre Hospital, Faculty of Health Sciences, Copenhagen University, Copenhagen, Denmark

**Keywords:** Patient-reported outcome measures, Tendinopathy, Psychometric properties, COSMIN

## Abstract

**Purpose:**

The evaluation of measurement properties such as reliability, measurement error, construct validity, and responsiveness provides information on the quality of the scale as a whole, rather than on an item level. We aimed to synthesize the measurement properties referring to reliability, measurement error, construct validity, and responsiveness of the Victorian Institute of Sport Assessment questionnaires (Achilles tendon—VISA-A, greater trochanteric pain syndrome—VISA-G, proximal hamstring tendinopathy—VISA-H, patellar tendon—VISA-P).

**Methods:**

A systematic review was conducted according to Consensus-based Standards for the Selection of Health Measurement Instruments methodology (COSMIN). PubMed, Cochrane, CINAHL, EMBASE, Web of Science, SportsDiscus, grey literature, and reference lists were searched. Studies assessing the measurement properties concerning reliability, validity, and responsiveness of the VISA questionnaires in patients with lower limb tendinopathies were included. Two reviewers assessed the methodological quality of studies assessing reliability, validity, and responsiveness using the COSMIN guidelines and the evidence for these measurement properties. A modified Grading of Recommendations Assessment Development and Evaluation (GRADE) approach was applied to the evidence synthesis.

**Results:**

There is moderate-quality evidence for sufficient VISA-A, VISA-G, and VISA-P reliability. There is moderate-quality evidence for sufficient VISA-G and VISA-P measurement error, and high-quality evidence for sufficient construct validity for all the VISA questionnaires. Furthermore, high-quality evidence exists with regard to VISA-A for sufficient responsiveness in patients with insertional Achilles tendinopathy following conservative interventions.

**Conclusions:**

Sufficient reliability, measurement error, construct validity and responsiveness were found for the VISA questionnaires with variable quality of evidence except for VISA-A which displayed insufficient measurement error.

**Level of evidence:**

IV.

**Registration details:**

Prospero (CRD42018107671); PROSPERO reference—CRD42019126595.

**Supplementary Information:**

The online version contains supplementary material available at 10.1007/s00167-021-06557-0.

## Introduction

The impact of lower limb tendinopathies on the patient, according to the International Scientific Tendinopathy Symposium Consensus from 2019, should be measured using validated outcome measures that can capture the core domains of the condition such as: functional testing, participation in life activities, psychological factors, physical function capacity, and most importantly disability via condition-specific patient-reported outcome measures (PROMs) [37, 59]. The Victorian Institute of Sport Assessment (VISA) questionnaires [4, 14, 51, 61] have been recommended by the consensus statement from 2019 [59] and are used globally in many different cultures, in research and clinical practice to assess the severity of symptoms and functional disability of patients with lower limb tendinopathies [30, 37, 58]. All four VISA are self-administered questionnaires, developed in English language, consisting of eight items, and assessing the severity of symptoms in patients with Achilles tendinopathy (VISA-A), greater trochanteric pain syndrome (VISA-G), proximal hamstring tendinopathy (VISA-H), and patellar tendinopathy (VISA-P) [4, 14, 51, 61]. Six out of eight items rate pain level during daily activities and functional tests, and two items provide information on the impact of tendinopathy in physical activity or sports participation. Scores are summed up with a score approaching 100 points representing a fully functional asymptomatic individual. The last item of the PROM (item 8) contributes significantly on the total score (may range from 0 to 30 out of 100 points), is divided into three parts, and inquires about sports participation or weight bearing activities (for patients with greater trochanteric pain syndrome). The participant must answer only one part depending on their symptom level and their interference with sports participation or weight-bearing activities.

In the first part of this systematic review [27], we evaluated the content and structural validity of all patient-reported VISA questionnaires (VISA-A, VISA-G, VISA-H, and VISA P). This systematic review showed variable results and that only very-low-quality evidence exists for the content validity and unidimensionality of VISA questionnaires when assessing the severity of symptoms and disability in patients with lower limb tendinopathies. In the second part of this systematic review, we aim to evaluate the rest of the measurement properties of patient-reported VISA questionnaires. This is important as VISA measurement properties, such as reliability, measurement error, construct validity, and responsiveness have been extensively evaluated in individual studies, since their development and publication without a systematic review, to our knowledge, to provide a comprehensive overview of the quality of these measurement properties. Unlike content and structural validity, the evaluation of these measurement properties provides information on the quality of the scale as a whole, rather than on an item level [48].

The foundation of evidence-based practice and thorough research is the use of outcome measures that are psychometrically sound. The validity and reliability, as well as the responsiveness of these measurement tools, is a prerequisite in making meaningful patient-centred clinical inferences. Thus, the aim of the present systematic review was to appraise and summarize the quality of the remaining measurement properties of VISA questionnaires: reliability, measurement error, construct validity, and responsiveness.

## Materials and methods

### Protocol registration

The search strategy and reporting of this systematic review followed the COnsensus-based Standards for the selection of health Measurement INstruments (COSMIN) methodology for systematic reviews of PROMs [48], the Cochrane group’s recommendations [20], and adhered to the Preferred Reporting Items for Systematic Reviews and Meta-Analyses (PRISMA) guidelines [42]. The protocol was prospectively registered in PROSPERO (CRD42019126595).

### Information sources and search methods

PubMed, Cochrane, CINAHL, EMBASE, Web of Science, and SportsDiscus databases were independently searched by two reviewers (AK and MS) from database inception to 19 May 2020 without language restriction.

Grey literature was searched via OpenGrey.eu, and the following registries: Clinical Trials.gov and EU clinical trials register. Reference lists, citation tracking results, and systematic reviews were also manually searched.

The search strategy included a comprehensive PROM filter developed by the COSMIN group [9, 56] and two basic strings of key terms (names of instruments and population of interest) (Online Resource 1).

### Study selection

The title and abstract of search results were independently screened by two authors (AK and MS) and full text of the remaining studies was checked against the criteria for eligibility. The reference lists of the included articles were also searched for additional potentially relevant studies [48]. A third author (VK) resolved disputes between the reviewers [31].

### Eligibility criteria

Studies were eligible if they were full-text articles in peer-reviewed journals, including patients with Achilles tendinopathy, greater trochanteric pain syndrome, proximal hamstring tendinopathy, or patellar tendinopathy and evaluating at least one of the measurement properties as defined by COSMIN taxonomy [44]: reliability, measurement error, construct validity (convergent and/or known groups), responsiveness, as well as interpretability and feasibility.

### Inclusion and exclusion criteria

The general inclusion criteria were: (a) all the types of studies assessing at least one measurement property of the VISA questionnaires (including development and not limited to validity, reliability, responsiveness, and interpretability); (b) including patients with Achilles tendinopathy, greater trochanteric pain syndrome, proximal hamstring tendinopathy, or patellar tendinopathy, as well as other groups of asymptomatic/injured individuals that were used in measurement properties assessment; and (c) only full-text articles in peer-reviewed journals. Following recommendations [48], we excluded studies that only used a VISA questionnaire as an outcome measurement instrument, for instance, randomized controlled trials, or studies in which a VISA was used in a validation study of another instrument; and criterion validity only was not an eligibility criterion due to the lack of an established gold standard for lower limb tendinopathies.

### Data extraction

Data from studies meeting the inclusion criteria were extracted by two reviewers (VK and AK) independently using standardized extraction forms and cross-checked. Any disagreements were resolved by consensus. We extracted publication details, sample size, patient and condition characteristics, details on PROM administration (setting, country, language, missing items, floor and ceiling effects, and completion time), data and indices for reliability, measurement error, convergent and divergent validity, and responsiveness. Furthermore, we extracted VISA scores of groups of individuals included in each study.

### Assessment of the methodological quality of single studies and evaluation of results against criteria for good measurement properties

The methodological quality of each eligible study on a measurement property was assessed separately using the COSMIN Risk of Bias checklist [43] and pre-formulated hypotheses as indicated by the COSMIN guidelines [9]. The development studies and the studies on measurement properties were assessed using COSMIN standards; boxes 6–10, including 8 items for reliability, 6 items for measurement error, 7 items for construct validity, and 13 items for responsiveness. Interpretability and feasibility (including ceiling and floor effects) are not formal measurement properties, because they do not refer to the quality of the PROM; thus, they were not evaluated; however, given that they are considered important aspects for the selection of a PROM, they were described in the systematic review [43].

Each standard and subsequently each study were rated as “very good”, “adequate”, “doubtful”, or “inadequate” quality. The methodological study quality score per measurement property was determined by the item with the lowest score (worse score counts) [48].

Subsequently, the results on each measurement property were rated against the updated criteria for good measurement properties [48, 55]. Each result was rated as “sufficient” (+), “insufficient” (−), or “indeterminate” (?). Two reviewers (AK and MS) independently rated the quality of measurement properties, while discrepancies were resolved by discussion with a third reviewer (VK).

### Rating the quality of evidence

Two reviewers (AK and MS) independently rated and summarized the quality of evidence for each measurement property using a modified GRADE approach, as suggested by the Cosmin guidelines [48]. Evidence was started at high quality and downgraded according to the presence and extent of specific dimensions recommended for the quality of evidence in PROM measurement properties studies: risk of bias (methodological quality), inconsistency (unexplained inconsistency of results across studies), imprecision (total sample size), and indirectness (evidence from population different than that of interest). The results were qualitatively summarized or quantitatively pooled (where applicable) and compared against the criteria for good measurement properties to determine whether the “overall” measurement property of the PROM is sufficient (+), insufficient (−), inconsistent (±), or indeterminate (?) [48]. To rate the pooled or qualitatively summarized results as sufficient or insufficient, the criterion of at least 75% consistent results had to be met [48].

### Statistical analysis

To our knowledge, there is no procedure yet defined for formal meta-analysis of intraclass correlation coefficient (ICC) values. To allow for description of an interpretable value of the pooled ICC coefficients, these raw values were pooled using the R statistical platform [49] (metafor package) [60] with the variance approximated as described in Noble et al. [46] using a random effects model. The uninterpretable Fisher *z*-transformed values are provided (Online Resource 2). Given the statistical heterogeneity observed (Cochrane’s Q statistic and I^2^), moderator analysis was conducted using subject groups (i.e., patients, asymptomatic subjects, mixed groups, and at-risk subjects). Values were presented as pooled mean estimate and 95% confidence intervals (CI).

For interpretability of sub-group (i.e., patients, at-risk, asymptomatic) VISA scores, standardized mean differences (SMD) and 95% CI were calculated from pooled weighted group scores to determine the magnitude of difference of the total score (Comprehensive Meta-Analysis software).

## Results

### Study characteristics

**Fig. 1:**
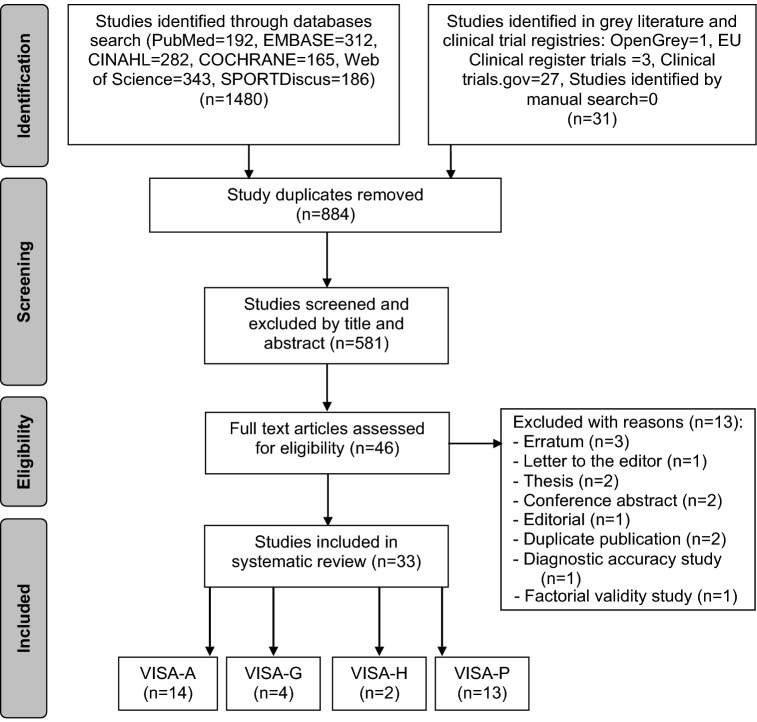
PRISMA flow diagram for study inclusion

Of the original 1511 studies, 34 remained after duplicate removal. Of these, 33 met the eligibility criteria appraising measurement properties of interest of this review (Fig. 1): VISA-A [10–12, 19, 21, 25, 26, 33, 35, 38, 40, 51, 53, 54], VISA-G [2, 13, 14, 22], VISA-H [4, 32], and VISA-P [1, 5, 15–18, 24, 28, 34, 39, 47, 61, 62, 64].

The review team decided that there is no gold standard for measuring pain, function, and sports participation in patients with lower limb tendinopathy; hence, the criterion validity was not evaluated in this review.

### Characteristics of the included study populations

Characteristics of the study population, condition, and details on instrument administration are presented in Table 1.

**Table 1:** Characteristics of the study population, condition, and details on instrument administration

Questionnaire	Population			Condition characteristics			Instrument administration		
	*n*	Age^a^	Gender ♀ (%)	Condition	Condition duration^b^	VISA score^c^	Setting	Country	Language
VISA-A									
Robinson et al. [51]	45	42.3 ± 11.4	40	AT (mixed)	21.0 ± 25.5 m (CI 7.7–23.1)	64.0 ± 17.0 (CI 59.0–69.0)	Clinic	Canada	English
Robinson et al. [51]	14	44.3 ± 14.8	43	sAT (mixed)	19.2 ± 4.1 m (CI 14.8–19.2)	44.0 ± 28.0 (CI 28.0–60.0)	Clinic	Canada	English
Robinson et al. [51]	63	23.0 ± 2.9	49	Controls	NA	96.0 ± 7.0 (CI 94.0–98.0)	University	Canada	English
Robinson et al. [51]	20	40.9 ± 9.1	45	At risk	NA	98.0 ± 3.0 (CI 97.0–99.0)	Running club	Canada	English
Silbernagel et al. [54]	51	43.1 ± 14.5 (CI 39.0–47.2)	63	AT (mixed)	31.8 ± 90.8 m (CI 6.3–57.4)	50.0 ± 23.0 (CI 44.0–56.0)	Clinic	Sweden	Swedish
Silbernagel et al. [54]	15	29.5 ± 4.3 (CI 27.1–31.9)	80	Controls	NA	96.0 ± 4.0 (CI 94.0–99.0)	NI	Sweden	Swedish
de Knikker et al. [10]	17	45.2 ± 9.9 (CI 40.1–50.3)	31	AT (mid-portion)	*Mdn* 13.0 w (IQR 34.0)	69.0 ± 16.7 (range 60.0–77.0)	Clinic	Netherlands	Dutch
de Knikker et al. [10]	20	35.4 ± 10.7 (CI 30.4–40.4)	55	Controls	NA	100.0 ± 1.5 (range 99.0–100)	Clinic	Netherlands	Dutch
Maffulli et al. [38]	50	Mean 26.4 (18–49)	NR	AT (mid-portion)	NR	51.8 ± 18.2	NI	Italy	Italian
Lohrer et al. [33]	15	44.6 ± 14.0 (CI 36.9–52.4)	NR	AT (mid-portion)	NR	73.1 ± 13.5 (CI 65.6–80.5)	Clinic	Germany	German
Lohrer et al. [33]	15	47.8 ± 11.4 (CI 41.5–54.1)	NR	sAT (mid-portion)	NR	44.9 ± 14.2 (CI 37.0–52.7)	Clinic	Germany	German
Lohrer et al. [33]	48	21.0 ± 3.9 (CI 20.0–22.1)	NR	Controls	NA	98.0 ± 7.1 (CI 95.9–100.0)	University	Germany	German
Lohrer et al. [33]	31	39.3 ± 11.7 (CI 35.0–43.6)	NR	At risk	NA	99.2 ± 2.0 (CI 98.5–99.9)	Running clubs	Germany	German
Lohrer et al. [35]	18	44.7 ± 13.3 (CI 38.1–51.4)	NR	HD	NR	62.6 ± 12.7 (CI 56.3–68.9)	Clinic	Germany	German
Lohrer et al. [35]	21	46.5 ± 12.7 (CI 40.8–52.3)	NR	sHD	NR	34.7 ± 18.3 (CI 26.4–43.0)	Clinic	Germany	German
Lohrer et al. [35]	48	21.0 ± 3.9 (CI 20.0–22.1)	NR	Controls	NA	98.0 ± 7.1 (CI 95.9–100.0)	University	Germany	German
Lohrer et al. [35]	31	39.3 ± 11.7 (CI 35.0–43.6)	NR	At risk	NA	99.2 ± 2.0 (CI 98.5–99.9)	Running clubs	Germany	German
Dogramaci et al. [12]	55	40.9 ± 6.2	29	AT (mixed)	14.2 ± 6.08 m	52.8 ± 13.9 (24.0–72.0)	Clinic	Turkey	Turkish
Dogramaci et al. [12]	55	38.5 ± 7.2	29	Controls	NA	97.1 ± 1.5 (95.0–100.0)	NI	Turkey	Turkish
McCormack et al. [40]	15	Mean range (52.7–53.5)	73	AT (insertional)	*Mean range* (16.3–23.2) w	*Mean range* (36.3–38.5)	Clinic	USA	English
Iversen et al. [21]	71	42.0 ± 13.0 (CI 39.0–45.0)	37	AT (mid-portion)	20.0 ± 20.0 m (CI 15.0–25.0)	51.0 ± 19.0 (CI 4.0–55.0)	Clinic	Denmark	Danish
Iversen et al. [21]	75	39.0 ± 13.0 (CI 36.0–42.0)	64	Controls	NA	93.0 ± 12.0 (CI 90.0–95.0)	Clinic	Denmark	Danish
Kaux et al. [25]	31	45.2 ± 15.2	23	AT (mixed)	NR	59.0 ± 18.0	Clinic	Belgium	French
Kaux et al. [25]	63	30.1 ± 10.7	29	Controls	NA	99.0 ± 1.0	University	Belgium	French
Kaux et al. [25]	22	29.1 ± 11	32	At risk	NA	94.0 ± 7.0	Sports clubs	Belgium	French
Hernandez-Sanchez et al. [19]	70	33.9 ± 12.0	51	AT (mixed)	12.1 ± 1.4 m	54.4 ± 12.6	Clinic & sport clubs	Spain	Spanish
Hernandez-Sanchez et al. [19]	70	20.3 ± 2.8	14	Controls	NA	98.1 ± 1.8	University	Spain	Spanish
Hernandez-Sanchez et al.[19]	70	24.1 ± 4.2	23	At risk	NA	92.6 ± 6.4	NI	Spain	Spanish
Keller et al. [26]	20	Mean 41.0 (25.0–49.0)	35	AT (mixed)	NR	*Mean* 67.16 (28.0–100.0)	Clinic	Chile	Chilean Spanish
Keller et al. [26]	20	Mean 43.0 (29.0–51.0)	30	AT-severe (mixed)	NR	*Mean* 24.7 (14.0–40.0)	Clinic	Chile	Chilean Spanish
Keller et al. [26]	20	Mean 38.0 (20.0–55.0)	50	Controls	NA	*Mean* 100.0	Clinic	Chile	Chilean Spanish
de Mesquita et al. [11]	39	31.2 ± 10.2	33	AT (mixed)	29.1 ± 39.8 m	63.1 ± 15.1	NI	Brazil	Brazilian Portuguese
de Mesquita et al. [11]	17	22.6 ± 4.2	41	Healthy	NA	95.2 ± 4.7	NI	Brazil	Brazilian Portuguese
de Mesquita et al. [11]	50	24.0 ± 4.7	38	At risk	NA	94.7 ± 5.3	NI	Brazil	Brazilian Portuguese
Sierevelt et al. [53]	104	48.5 ± 11.6	47	AT (mixed)	NR	52.4 ± 19.7_athletes_ 22.0 ± 15.7	Clinic	Netherlands	Dutch
Fearon et al. [14]	52	58.9 ± 13.64♀ 53.0 ± 15.13♂	90	GTPS	NR	47.00 (42.62–50.18)	Clinic	Australia	English
Fearon et al. [14]	31	57.4 ± 5.59♀ 58.4 ± 5.22♂	77	Controls	NA	99.84 (99.60–100.00)	Clinic	Australia	English
Ebert et al. [13]	56	65.8 ± 7.8 (51–84)	93	HATR	3.9 ± 3.7 yr (0.5–20)	43.0 ± 15.0	Clinic	Australia	English
Beaudart et al. [2]	52	Mdn 59.5 (IQR 42.2–66.0)	75	GTPS	NR	*Mdn* 60.5 (IQR 43–71)	Clinic	Belgium, France	French
Beaudart et al. [2]	54	Mdn 42 (IQR 24.0–58.2)	48	Controls	NA	*Mdn* 100 (IQR 100–100)	Clinic	Belgium, France	French
Jorgensen et al. [22]	49	56.0 ± 10.2	96	GTPS	NR	61.94 ± 5.78 (48–77)	Clinic	Denmark	Danish
Jorgensen et al. [22]	58	50.0 ± 8.9	71	Controls	NA	98.0 ± 4.05 (86–100)	Clinic	Denmark	Danish
VISA-H									
Cacchio et al. [4]	20	Mean 23.7 (18–25)	30	nsPHT	NR	56.7 ± 11.6	Clinic	Italy	English
Cacchio et al. [4]	10	Mean 21.4 (18–23)	20	sPHT	NR	45.8 ± 12.2	Clinic	Italy	English
Cacchio et al. [4]	30	Mean 23.1 (18–26)	33	Controls	NA	99.3 ± 1.2	Clinic	Italy	English
Locquet et al. 2[32]	16	32.4 ± 12.0	35	PHT	NR	*Mdn* 58 (IQR 37.75–73.0)	NI	Belgium	French
Locquet et al. [32]	15			Controls	NA	*Mdn* 100 (IQR 95.0–100.0)	NI	Belgium	French
Locquet et al. [32]	20			At risk	NA	*Mdn* 97 (IQR 34.0–100.0)	NI	Belgium	French
VISA-P									
Visentini et al. [61]	14	25.0 ± 6.0	NR	PT	NR	55.0 ± 12.0	Clinic	Australia	English
Visentini et al. [61]	26	31.0 ± 9.0	NR	Controls	NA	95.0 ± 8.0	University	Australia	English
Visentini et al. [61]	15	31.0 ± 9.0	NR	Pre-surgical PT	NR	22.0 ± 17.0	Clinic	Australia	English
Visentini et al. [61]	100	24.0 ± 6.0	NR	At risk	NA	93.0 ± 11.0	University	Australia	English
Visentini et al. [61]	26	27.0 ± 7.0	NR	Other MSK conditions	NA	92.0 ± 13.0	Clinic	Australia	English
Frohm et al. [15]	17	22.0 ± 5.0	0	PT	NR	47.8 ± 20.3	Sports centre	Sweden	Swedish
Frohm et al. [15]	17	24.0 ± 6.0	53	Controls	NA	83.1 ± 12.6	Sports centre	Sweden	Swedish
Frohm et al. [15]	17	26.0 ± 3.0	0	At risk	NA	79.0 ± 24.2	Sports centre	Sweden	Swedish
Maffulli et al. [39]	25	Mean 27.9 (18–32)	0	PT	NR	*Mean* 44.3 (33–61)	Clinic	Italy	Italian
Zwerver et al. [64]	14	25.1 ± 3.7	21	PT	NR	58.2 ± 18.9	Clinic	Netherlands	Dutch
Zwerver et al. [64]	18	20.0 ± 1.5	61	Controls	NA	95.3 ± 8.8	NI	Netherlands	Dutch
Zwerver et al. [64]	15	25.2 ± 4.7	47	At risk	NA	88.6 ± 11.1	NI	Netherlands	Dutch
Zwerver et al. [64]	19	19.2 ± 1.2	79	Other MSK conditions	NR	76.6 ± 24.3	NI	Netherlands	Dutch
Zwerver et al. [64]	17	24.7 ± 4.5	35	Other knee injuries	NR	61.9 ± 24.1	NI	Netherlands	Dutch
Hernandez-Sanchez et al. [17]	40	24.4 ± 5.1	10	PT	17.7 ± 17.1 m	54.8 ± 13.2	Clinic	Spain	Spanish
Hernandez-Sanchez et al. [17]	40	21.3 ± 3.1	2.5	Controls	NA	95.4 ± 2.5	University	Spain	Spanish
Hernandez-Sanchez et al. [17]	40	24.5 ± 4.5	20	At risk	NA	90.0 ± 9.7	NI	Spain	Spanish
Hernandez-Sanchez et al. [17]	30	24.1 ± 4.2	23	Other knee injuries	NR	56.4 ± 11.3	Clinic	Spain	Spanish
Lohrer et al. [34]	23	34.8 ± 13.1	NR	PT	NR	62.3 ± 13.0	Clinic	Germany	German
Lohrer et al. [34]	32	24.8 ± 1.8	NR	Controls	NA	96.0 ± 5.6	University	Germany	German
Lohrer et al. [34]	25	38.7 ± 8.1	NR	At risk	NA	92.7 ± 6.9	Training clubs	Germany	German
Park et al. [47]	23	15.9 ± 1.9	53.5	PT	NR	67.6 ± 15.7	NI	Korea	Korean
Park et al. [47]	5			Controls	NA	92.6 ± 8.6	NI	Korea	Korean
Wageck et al. [62]	52	23.4 ± 6.8	27	PT	NR	59.1 ± 17.5	Clinic & training clubs	Brazil	Brazilian Portuguese
Hernandez-Sanchez et al. [18]	90	25.9 ± 5.4	22	PT	14.1 ± 13.9 m	50.1 ± 18.4	Clinic	Spain	Spanish
Korakakis et al. [28]	32	25.5 ± 4.4	40	PT	NR	53.3 ± 8.1 (35–66)	Clinic	Greece	Greek
Korakakis et al. [28]	61	28.9 ± 6.1	64	Controls	NA	95.0 ± 6.7 (78–100)	Training clubs	Greece	Greek
Korakakis et al. [28]	64	24.3 ± 5.2	41	At risk	NA	97.9 ± 3.7 (78–100)	Training clubs	Greece	Greek
Korakakis et al.[28]	30	26.4 ± 4.6	43	Other knee injuries	NR	60.1 ± 6.8 (47–72)	Clinic	Greece	Greek
Celebi et al. [5]	34	21.8 ± 5.8	41	PT	NR	58.8 ± 12.1	Clinic	Turkey	Turkish
Celebi et al. [5]	31	24.3 ± 3.6	45	Controls	NA	93.7 ± 8.9	Clinic	Turkey	Turkish
Celebi et al. [5]	24	28.1 ± 5.4	33	At risk	NA	81.1 ± 13.7	Clinic	Turkey	Turkish
Kaux et al. [24]	28	29.1 ± 8.6	7	PT	NR	53.0 ± 17.0	NI	Belgium	French
Kaux et al. [24]	22	31 ± 13.5	36	Controls	NA	99.0 ± 2.0	NI	Belgium	French
Kaux et al. [24]	42	26.3 ± 6.9	38	At risk	NA	86.0 ± 14.0	NI	Belgium	French
Hernandez-Sanchez et al. [16]	249	27.5 ± 7.8♀ 30.2 ± 8.2♂	41	PT	NR	46.5 ± 17.1♀ 46.0 ± 17.3♂	Clinic & training clubs	Spain	Spanish
Acharya et al. [1]	35	18.9 ± 2.2	NR	PT	NR	NR	NI	India	Kannada
Acharya et al. [1]	35	19.0 ± 1.1	NR	Controls	NA	NR	NI	India	Kannada

*AT* Achilles tendinopathy, *CI* 95% confidence intervals, *controls* asymptomatic individuals, *GTPS* greater trochanteric pain syndrome, *HATR* hip abductor tendons reattachment, *HD* Haglund’s disease, *IQR* interquartile range, *m* months, *Mdn* median, *NA* not applicable, *NI* no information, *NR* not reported, *ns* non-surgical, *PT* patellar tendinopathy, *s* surgical, *SD* standard deviation, *w* weeks, *yr* years

^a^Age in mean ± SD (range), unless stated otherwise

^b^Condition duration in mean ± SD (range), unless stated otherwise

^c^VISA score in mean ± SD (range), unless stated otherwise

### Quality, results, and evidence synthesis of studies evaluating reliability

#### VISA-A

Thirteen studies [10–12, 19, 21, 25, 26, 33, 35, 38, 51, 53, 54] assessed the reliability of the VISA-A in 907 patients and asymptomatic individuals. All summarized studies presented results of sufficient reliability ranging from 0.79 to 0.993 except two studies, where the reliability coefficients did not meet the criteria of ICC > 0.70. The treatment provided in Achilles tendinopathy patients [10] and the continuation of running in the “at-risk” group [33] during the test–retest period may explain these inconsistencies (Table 2).

**Table 2:** Quality assessment and results of studies evaluating reliability, measurement error, hypotheses for construct validity, and responsiveness of VISA questionnaire studies

	Country (language)	Reliability			Measurement error			Hypotheses testing			Responsiveness		
		*n*	COSMIN quality rating	Result (rating)	*n*	COSMIN quality rating	Result (rating)	*n*	COSMIN quality rating	Result (rating)	*n*	COSMIN quality rating	Result (rating)
VISA-A													
Robinson et al. [51]	Canada (English)	45	Doubtful	Pearson’s *r* = 0.81 patients (?)	NT			45	Inadequate^a^	In line with 2^a^ hypo’s (2 +)	NT		
		12	Doubtful	Pearson’s * r* = 0.98 healthy (?)				142	Very good^b^	In line with 6^b^ hypo’s (6 +)			
Silbernagel et al. [54]	Sweden (Swedish)	22	Inadequate	ICC = 0.89 patients ( +)	NT			66	Inadequate^a^	In line with 1^a^ hypo (1 +)	NT		
		15	Doubtful	ICC = 0.90 healthy ( +)					Very good^b^	In line with 1^b^ hypo (1 +)			
de Knikker et al. [10]	Netherlands (Dutch)	17	Doubtful	ICC = 0.60 (0.19–0.84) patients (−)	17	Doubtful	SEM = 7.0 SDC_95_ = 19.0 LoA (− 26.49 to 32.72) (−)	17	Inadequate^a^	In line with 2^a^ hypo’s (2 +) Not in line with 1^a^ hypo (1−)	NT		
		20	Doubtful	ICC = 0.87 (0.71–0.95) healthy ( +)					Very good^b^	In line with 1^b^ hypo (1 +)			
Maffulli et al. [38]	Italy (Italian)	50	Inadequate	Pearson’s r NR (?)	NT			NT			NT		
Lohrer et al. [33]	Germany (German)	15	Doubtful	ICC = 0.87 patients ( +)	NT			109	Inadequate^a^	In line with 2^a^ hypo’s (2 +)	NT		
		48	Doubtful	ICC = 0.97 healthy ( +)					Very good^b^	In line with 6^b^ hypo’s (6 +)			
		31	Doubtful	ICC = 0.60 at risk (−)									
Lohrer et al. [35]*	Germany (German)	18	Doubtful	ICC = 0.96 patients ( +)	NT			118	Inadequate^a^	In line with 2^a^ hypo’s (2 +)	NT		
		48	Doubtful	ICC = 0.97 healthy ( +)					Very good^b^	In line with 7^b^ hypo’s (7 +)			
		31	Doubtful	ICC = 0.60 at risk (−)									
Dogramaci et al. [12]	Turkey (Turkish)	52	Doubtful	Pearson’s * r* = 0.99 mixed (?)	NT			110	Adequate^a^	In line with 1^a^ hypo’s (1 +) Not in line with 3^a^ hypo (3−)	NT		
									Inadequate^a^	In line with 1^a^ hypo’s (1 +)			
									Very good^b^	In line with 1^b^ hypo’s (1 +)			
McCormack et al. [40]	USA (English)	NT			NT			NT			15	Very good^d^	AUC = 0.94 (0.85 to 1.0) MIC = 6.5 ( +)
Iversen et al. [21]	Denmark (Danish)	36	Doubtful	ICC = 0.79 patients ( +)	NT			146	Very good^b^	In line with 1^b^ hypo’s (1 +)	28	Inadequate^d^	In line with 1 hypo’s (1 +)^d^
		75	Doubtful	ICC = 0.97 healthy ( +)									
Kaux et al. [25]	Belgium (French)	31	Inadequate	ICC = 0.99 (0.996–0.998) patients ( +)	NT			99	Adequate^a^	In line with 6^a^ hypo’s (6 +) Not in line with 2^a^ hypo (2−)	NT		
								116	Very good^b^	In line with 2^b^ hypo’s (2 +) Not in line with 1^b^ hypo’s (1−)			
Hernandez-Sanchez et al. [19]	Spain (Spanish)	210	Doubtful	ICC = 0.993 (0.991–0.995) mixed ( +)	210	Doubtful	SEM = 2.53 SDC_95_ = 7.0 LoA (− 5.9 to 4.64) ( +)	70	Adequate^a^	In line with 6^a^ hypo’s (6 +) Not in line with 2^a^ hypo (2−)	70	Adequate^c^	In line with 2 hypo’s (2 +)^c^
								210	Very good^b^	In line with 3^b^ hypo’s (3 +)		Inadequate^d^	ES = 2.165 SRM = 1.923 ( +)
Keller et al. [26]	Chile (Chilean Spanish)	40	Doubtful	Pearson’s * r* = 0.84 Spearman’s rho = 0.837 patients (?)	NT			60	Doubtful^b^	In line with 3^b^ hypo’s (3 +)	NT		
de Mesquita et al. [11]	Brazil (Brazilian Portuguese)	39	Doubtful	ICC = 0.84 (0.71–0.91) patients ( +)	39	Doubtful	SEM = 3.25 SDC_95_ = 9.02 (−)	106	Adequate^a^	In line with 6^a^ hypo’s (6 +)	NT		
Sierevelt et al. [53]	Netherland (Dutch)	52	Doubtful	ICC = 0.97 (0.95–0.98) patients ( +)	52	Doubtful	SEM = 4.07 SDC_95_ = 11.28 (−)	93	Adequate^a^	In line with 16^a^ hypo’s (16 +) Not in line with 2^a^ hypo (2−)	NT		
Pooled or summary result (overall rating)		708	Sufficient reliability ( +) Pooled ICC = 0.918 (0.874–0.961)^†^		318	Insufficient measurement error (−) Weighted SEM average 3.1 (range 2.52–7.0) Weighted SDC average 8.6 (range 7.0–19.0)		715	Sufficient construct validity^a^ ( +) / 43 + and 10 − (81.1%)		70	Sufficient responsiveness ( +)^c^	
		440	Pooled ICC = 0.911 (0.847–0.975)^‡^ ( +)					976	Sufficient construct validity^b^ ( +) / 24 + and 1 − (96.9%)		113	Sufficient responsiveness AUC = 0.94 and hypotheses ( +)^d^	
VISA-G													
Fearon et al. [14]	Australia (English)	26	Doubtful	ICC = 0.827 (0.638–0.923) patients ( +)	26	Doubtful	SEM = 1.883 SDC_95_ = 5.2** ( +)	83	Adequate^a^	In line with 3^a^ hypo’s (3 +) Not in line with 1^a^ hypo’s (1−)	NT		
									Very good^b^	In line with 1^b^ hypo’s (1 +)			
Ebert et al. [13]	Australia (English)	ΝΤ			NT			NT			56	Adequate^c^	In line with 2 hypo’s (2 +)^c^
												Very good^d^	AUC = 0.70 ( +) (0.56–0.81)^d^ MIC = 29 points^d^
Beaudart et al. [2]	Belgium, France (French)	106	Inadequate	ICC = 0.99 (0.99–0.99) mixed ( +)	106	Inadequate	SEM = 1.64 SDC_95_ = 4.55 ( +)	106	Adequate^a^	In line with 6^a^ hypo’s (6 +) Not in line with 2^a^ hypo’s (2 −)	NT		
									Very good^b^	In line with 1^b^ hypo’s (1 +)			
Jorgensen et al. [22]	Denmark (Danish)	49	Doubtful	ICC = 0.96 (0.93–0.98) patients ( +)	107	Doubtful	SEM = 0.6 SDC_95_ = 3.17 LoA NR ( +)	NT			NT		
		58	Doubtful	ICC = 0.98 (0.97–0.99) healthy ( +)									
Pooled or summary result (overall rating)		239	Sufficient reliability ( +) ICC ranged 0.827–0.99		239	Sufficient measurement error ( +) Weighted SEM average 1.2 (range 0.6–1.88) Weighted SDC average 4.0 (range 3.17–5.2)		189	Sufficient construct validity^a^ ( +) / 9 + and 3− (75.0%)		56	Sufficient responsiveness ( +) AUC ≥ 0.70 and hypotheses +	
									Sufficient construct validity^b^ ( +) / 2 + and 0− (100%)				
VISA-H													
Cacchio et al. [4]	Italy (English)	16	Inadequate	ICC = 0.92 (0.80–0.97) patients ( +)	16	Inadequate	SEM = 1.35 SDC_95_ = 3.7** patients ( +)	25	Inadequate^a^	In line with 4^a^ hypo’s (4 +)	55	Inadequate^c^	In line with 2 hypo’s (2 +)^c^
		9	Inadequate	ICC = 0.90 (0.63–0.97) surgical ( +)	9	Inadequate	SEM = 1.56 MDC_95_ = 4.3** surgical ( +)	55	Very good^b^	In line with 3^b^ hypo’s (3 +)	16	Very good^d^	AUC = 0.90^d^ ( +) MIC = 22 points^d^ ES = 2.2 SRM = 1.6 patients ES = 3.3 SRM = 2.2 surgical group
		30	Inadequate	ICC = 0.95 (0.90–0.97) healthy ( +)	30	Inadequate	SEM = 0.25 SDC_95_ = 0.7** healthy ( +)						
Locquet et al. [32]	Belgium (French)	16	Inadequate	ICC = 0.916 (0.80–0.966) patients ( +)	NT			51	Adequate^a^	In line with 6^a^ hypo’s (6 +) Not in line with 2^a^ hypo’s (2−)	NT		
		51	Inadequate	ICC = 0.993 (0.988–0.996) mixed ( +)					Doubtful^b^	In line with 3^b^ hypo’s (3 +)			
Pooled or summary result (overall rating)		106	Sufficient reliability ( +) ICC ranged 0.90–0.993		55	Sufficient measurement error ( +) SEM range 0.25–1.56 SDC range 0.7–4.3		106	Sufficient construct validity^a^ ( +) / 10 + and 2 − (83.3%)		55	Sufficient responsiveness ( +) AUC = 0.90 and hypotheses +	
									Sufficient construct validity^b^ ( +) / 6 + and 0 − (100%)				
VISA-P													
Visentini et al. [61]	Australia (English)	9	Doubtful	Pearson’s *r* = 0.87 patients (?)	NT			81	Inadequate^a^	In line with 4^a^ hypo’s (4 +)	15	Inadequate^c^	In line with 2 hypo’s (2 +)^c^
								155	Adequate^b^	In line with 6^b^ hypo’s (6 +)		Inadequate^d^	In line with 2 hypo’s (2 +)^d^
Frohm et al. [15]	Sweden (Swedish)	51	Doubtful	ICC = 0.97 mixed ( +)	51	Doubtful	LoA NR	51	Very good^b^	In line with 2^b^ hypo’s (2 +)	NT		
Maffulli et al. [39]	Italy (Italian)	25	Inadequate	Pearson’s *r* NR (?)	NT			NT			NT		
Zwerver et al. [64]	Netherland (Dutch)	71	Inadequate	ICC = 0.74 healthy ( +)	71	Inadequate	LoA NR	89	Very good^b^	In line with 3^b^ hypo’s (3 +) Not line with 2^b^ hypo’s (2−)	NT		
Hernandez-Sanchez et al. [17]	Spain (Spanish)	150	Doubtful	ICC = 0.994 (0.992–0.996) mixed ( +)	150	Doubtful	LoA (− 6.8 to 6.0) mixed ( +)	150	Adequate^a^	In line with 9^a^ hypo’s (9 +) Not in line with 1^a^ hypo’s (1 −)	40	Inadequate^d^	In line with 1 hypo’s (1 +)^d^ ES = 1.14 SRM = 1.17 ( +)
									Very good^b^	In line with 5^b^ hypo’s (5 +) Not in line with 1^b^ hypo’s (1 −)			
Lohrer et al. [34]	Germany (German)	23	Doubtful	ICC = 0.878 patients ( +)	23	Doubtful	SEM = 4.54 SDC_95_ = 12.6 patients ( +)	80	Inadequate^a^	In line with 2^a^ hypo’s (2 +)	NT		
		57	Doubtful	ICC = 0.872 healthy ( +)	57	Doubtful	SEM = 2.25 SDC_95_ = 6.2 healthy ( +)		Very good^b^	In line with 4^b^ hypo’s (4 +)			
Park et al. [47]	Korea (Korean)	28	Doubtful	ICC = 0.96 mixed ( +)	NT			28	Doubtful^b^	In line with 1^b^ hypo’s (1 +)	NT		
Wageck et al. [62]	Brazil (Brazilian Portuguese)	52	Inadequate	ICC = 0.91 (0.85–0.95) patients ( +)	52	Inadequate	SEM = 5.2 SDC_95_ = 14.4** patients ( +)	52	Adequate^a^	In line with 1^a^ hypo’s (1 +)	32	Inadequate^d^	In line with 1 hypo’s (1 +)^d^ ES = 0.97 ( +)
Hernandez-Sanchez et al. [18]	Spain (Spanish)	90	Inadequate	ICC = 0.95 (0.93–0.97) patients ( +)	90	Inadequate	SEM = 4.0 SDC_95_ = 11.1* patients ( +)	NT			90	Very good^c^	In line with 1 hypo’s (1 +)^c^
												Doubtful^d^	AUC = 0.924 (0.848–0.969) MIC = 16.0 ± 4.7 ( +)
Korakakis et al. [28]	Greece (Greek)	187	Doubtful	ICC = 0.82 (0.76–0.86) mixed ( +)	32	Doubtful	SEM = 3.46 SDC_95_ = 9.6 patients ( +)	187	Inadequate^a^	In line with 1^a^ hypo’s (1 +)	NT		
					64	Doubtful	SEM = 1.58 SDC_95_ = 4.38 at risk ( +)						
					61	Doubtful	SEM = 2.86 SDC_95_ = 7.93 healthy ( +)		Very good^b^	In line with 6^b^ hypo’s (6 +)			
					187	Doubtful	LoA (−3.9 to 4.1) mixed ( +)						
Celebi et al. [5]	Turkey (Turkish)	89	Inadequate	ICC = 0.96 mixed ( +)	NT			89	Very good^b^	In line with 2^b^ hypo’s (2 +)	NT		
Kaux et al. [24]	Belgium (French)	28	Inadequate	ICC = 0.99 (0.996–0.999) patients ( +)	28	Inadequate	SEM = 0.522 SDC_95_ = 1.446 patients ( +)	85	Adequate^a^	In line with 7^a^ hypo’s (7 +) Not in line with 1^a^ hypo’s (1−)	NT		
								92	Very good^b^	In line with 2^b^ hypo’s (2 +)			
Acharya et al. [1]	India (Kannada)	35	Doubtful	ICC = 0.97 (0.95–0.98) patients ( +)	NT			70	Inadequate^a^	In line with 1^a^ hypo’s (1 +)	NT		
		35	Doubtful	ICC = 0.96 (0.94–0.98) healthy ( +)									
Pooled or summary result (overall rating)		896	Sufficient reliability ( +) Pooled ICC = 0.964 (0.948–0.980)^†^		587	Sufficient measurement error ( +) Weighted SEM average 3.82 (range 0.52–5.2) patients Weighted SDC average 10.6 (range 1.4–14.4) patients		705	Sufficient construct validity^a^ ( +) / 25 + and 2− (92.6%)		105	Sufficient responsiveness ( +)^c^	
		733	Pooled ICC = 0.970 (0.955–0.986)^‡^ ( +)					921	Sufficient construct validity^b^ ( +) / 31 + and 3− (91.2%)		177	Sufficient responsiveness AUC = 0.924 and hypotheses ( +)^d^	
		228	Pooled ICC = 0.961 (0.932–0.991)^§^ ( +)										

Values are presented as “value (95% confidence intervals)” unless stated otherwise

*AUC* area under the curve, *ES* effect size, *hypo’s* hypotheses, *ICC* intraclass correlation coefficient, *LoA* limits of agreement, *MIC* minimally important change, *NR* not reported, *NT* not tested, *SDC* smallest detectible change, *SEM* standard error of measurement, *SRM* standardised response mean

*The study validated VISA-A in a population with different pathology

**SDC calculated using equation provided by Terwee et al.[55]

^a^Comparisons with other outcome measurement instruments for construct validity

^b^Known group’s validity

^c^Construct approach: hypotheses testing; comparison with other outcome measurement instruments

^d^Construct approach: hypotheses testing; before and after intervention

^†^Pooled coefficient in a mixed population of patients, asymptomatic controls, and at-risk individuals

^‡^Pooled coefficient in population including patients

^§^Pooled coefficient in patients

**Fig. 2:**
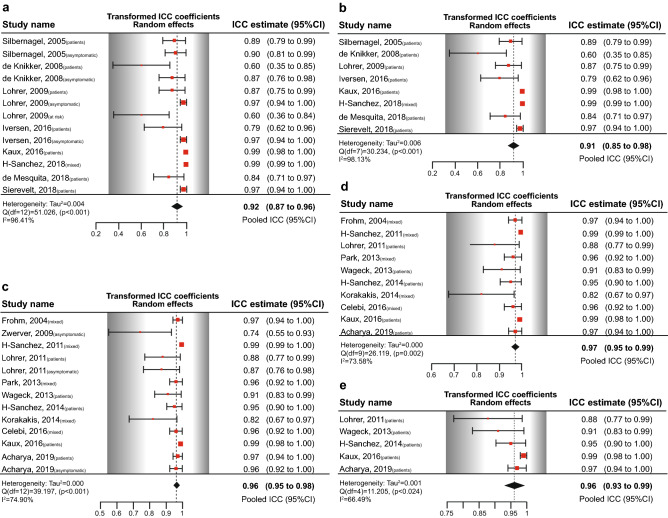
Forest plots of pooled ICC coefficients for the Victorian Institute of Sport Assessment scale—Achilles (VISA-A) and Patella (VISA-P). **a** Pooled ICC coefficients from all studies evaluated VISA-A, **b** pooled ICC coefficients for VISA-A studies including patients in the sample (only patients or mixed with asymptomatic individuals), **c** pooled ICC coefficients from all studies evaluated VISA-P, **d** pooled ICC coefficients for VISA-P studies including patients in the sample (only patients or mixed with asymptomatic individuals), and **e** pooled ICC coefficients for VISA-P studies including only patients in the sample. *CI* confidence intervals, *ICC* intraclass correlation coefficient, *mixed* mixed sample of participants and asymptomatic individuals

The pooled ICC coefficient was 0.918 (Fig. 2a). By subgrouping the studies that included patients (only, or mixed group of patients and asymptomatic individuals), the pooled estimate for ICC was 0.911 (Fig. 2b). Moderator analysis did not meaningfully alter the pooled estimate (ICC = 0.914, 95% CI 0.809–1.00, *I*^2^ = 95.79%).

There are very-low- and moderate-quality evidences for sufficient reliability of VISA-A in a mixed population of patients, asymptomatic and at-risk individuals and in patients with Achilles tendinopathy, respectively (Table 3).

**Table 3:** Evidence synthesis of the measurement properties of the Victorian Institute of Sport Assessment questionnaire (VISA) questionnaires to measure pain and physical functioning in patients with lower limb tendinopathies

PROM	Reliability		Measurement error		Hypotheses testing for construct validity		Responsiveness	
	Rating of results	Quality of evidence	Rating of results	Quality of evidence	Rating of results	Quality of evidence	Rating of results	Quality of evidence
VISA-A	+	Very low^a,b,d†^	−	Moderate^a^	+	High^e,f^	+	Low^a,b,g^
	+	Moderate^a,‡^					+	High^h^
VISA-G	+	Moderate^a^	+	Moderate^a^	+	High^e,f^	+	Very low^a,b,c,g^
							+	Low^b,c,h^
VISA-H	+	Low^a^	+	Very low^a,b^	+	Moderate^a,e^	+	Very low^a,b,g^
					+	High^f^	+	Moderate^b,h^
VISA-P	+	Low^a,c,†^	+	Moderate^a^	+	High^e,f^	+	High^g^
	+	Low^a,c,‡^					+	Low^a,h^
	+	Moderate^a,§^						

*PROMs* patient-reported outcome measures, *(*+*)* sufficient results, *(−)* insufficient results

^a^Risk of bias: (most studies of doubtful quality, or only one study of adequate quality, or multiple studies of inadequate quality, or only one inadequate study)

^b^Imprecision: sample size < 100

^c^Indirectness: patients had symptomatic partial or full thickness tears of gluteus minimus, along with the anterior portion of gluteus medius, or only part of population consists of patients

^d^Inconsistency: inconsistent results based on quality criteria

^e^Convergent validity

^f^Known group’s validity

^g^Construct approach: hypotheses testing; comparison with other outcome measurement instruments

^h^Construct approach: hypotheses testing; before and after intervention

^†^Pooled coefficient in a mixed population of patients, asymptomatic controls, and at-risk individuals

^‡^Pooled coefficient in population including patients

^§^Pooled coefficient in patients

#### VISA-G

Three studies [2, 14, 22] assessed the reliability of the VISA-G in 239 patients and asymptomatic individuals (Table 2).

There is moderate-quality evidence for sufficient reliability of VISA-G with ICC values ranging from 0.827 to 0.99 (Table 3).

#### VISA-H

Two studies [4, 32] assessed the reliability of the VISA-H in 106 patients and asymptomatic individuals (Table 2).

There is low-quality evidence for sufficient reliability of VISA-G ranging from 0.90 to 0.993 (Table 3).

#### VISA-P

Thirteen studies [1, 5, 15, 17, 18, 24, 28, 34, 39, 47, 61, 62, 64] assessed the reliability of the VISA-P in 930 patients with patellar tendinopathy and asymptomatic individuals. All summarized studies presented results of sufficient reliability ranging from 0.74 to 0.994 except two studies that the reliability coefficients did not meet the criteria of an ICC > 0.70 (Table 2).

The pooled ICC coefficient was 0.964 (Fig. 2c). By subgrouping the studies that included only patients with patellar tendinopathy or a mixed group of individuals including patients the pooled estimates for ICC were 0.970 and 0.961, respectively (Fig. 2d, e). Moderator analysis did not meaningfully alter the pooled estimate (ICC = 0.979, 95% CI 0.931–1.00, *I*^2^ = 66.89%).

There is low- and moderate-quality evidence for sufficient reliability of VISA-P in mixed populations and in patients with patellar tendinopathy only, respectively (Table 3).

### Quality, results, and evidence synthesis of studies evaluating measurement error

#### VISA-A

Four cross-cultural adaptations [10, 11, 19, 53] assessed the measurement error of the VISA-A in 318 patients and asymptomatic individuals (Table 2).

There is moderate-quality evidence for insufficient measurement error of the VISA-A with standard error of measurement (SEM) and smallest detectable change (SDC) values ranging from 2.53 to 7.0 and 7.0 to 19.0 points, respectively (Table 3).

#### VISA-G

Three studies [2, 14, 22] assessed the measurement error of the VISA-G in 239 patients and asymptomatic individuals (Table 2).

There is moderate-quality evidence for sufficient measurement error of VISA-G with SEM and SDC values ranging from 0.6 to 1.883 and 3.17 to 5.2 points, respectively (Table 3).

#### VISA-H

Only the development study [4] assessed the measurement error of the VISA-H in 55 patients with proximal hamstring tendinopathy and asymptomatic individuals (Table 2).

There is very-low-quality evidence for sufficient measurement error of VISA-H with SEM and SDC values ranging from 0.25 to 1.56 and 0.7 to 4.3 points, respectively (Table 3).

#### VISA-P

Eight studies [15, 17, 18, 24, 28, 34, 62, 64] assessed the measurement error of the VISA-P in 587 patients with patellar tendinopathy and asymptomatic individuals (Table 2).

There is moderate-quality evidence for sufficient measurement error of the VISA-P with SEM and SDC values ranging from 0.522 to 5.2 and 1.446 to 14.4 points, respectively (Table 3).

### Quality, results, and evidence synthesis of studies evaluating hypotheses for construct validity

#### VISA-A

Eleven studies [10–12, 19, 21, 25, 26, 35, 51, 53, 54] assessed construct validity using as comparators generic tendon grading systems, valid and reliable lower limb PROMs (i.e., Orthopaedic Foot and Ankle Society, Foot and Ankle Outcome Score questionnaire), or generic measures of health status (i.e., the Medical Outcomes Study 36-Item Short-Form Health Survey—SF36). In addition, assessed known group’s validity by comparing the scores of patients, asymptomatic, or “at-risk” for tendinopathy individuals (Table 2).

There is high-quality evidence for sufficient hypotheses testing for construct validity of the VISA-A from consistent findings (Table 3).

#### VISA-G

Two studies [2, 14] assessed known groups and convergent validity using as comparator instruments the Harris Hip Score, the Oswestry Disability Index, and the Short Form 36 or comparing the VISA-G scores between patients and asymptomatic individuals (Table 2).

There is high-quality evidence for sufficient hypotheses testing for construct validity (convergent and known groups) of VISA-G from consistent findings (Table 3).

#### VISA-H

Two studies [4, 32] assessed construct and known group’s validity of the VISA-H in 106 patients and asymptomatic individuals (Table 2).

There is moderate- and high-quality evidence for sufficient hypotheses testing of VISA-H for convergent and known group’s validity, respectively (Table 3).

#### VISA-P

Eleven studies [1, 5, 15, 17, 24, 28, 34, 47, 61, 62, 64] assessed construct validity using as comparators generic tendon grading systems (i.e., Nirchl pain scale, Blazina classification system), valid and reliable lower limb PROMs (i.e., Lysholm questionnaire, Cincinnati knee scale, and Kujala scoring questionnaire), or generic measures of health status (i.e., SF36), as well as assessed known group’s validity by comparing the scores of patients, asymptomatic, or “at-risk” for tendinopathy individuals (Table 2).

There is high-quality evidence for sufficient hypotheses testing for construct validity (convergent and known groups) of the VISA-P from consistent findings (Table 3).

### Quality, results, and evidence synthesis of studies evaluating responsiveness

#### VISA-A

Three studies using the construct approach tested hypotheses for responsiveness by comparing the VISA-A change scores with the SF-36 [19] or by assessing the effect magnitude of an intervention in patients with Achilles tendinopathy [19, 21, 40] (Table 2).

There is low-quality evidence for sufficient responsiveness of the VISA-A as compared with SF-36, and high-quality evidence for sufficient responsiveness following rehabilitation with a minimally important change (MIC) of 6.5 points (Table 3).

#### VISA-G

One study [13] tested hypotheses for responsiveness by comparing the VISA-G change scores with the Oxford Hip Score and the Harris Hip Score, or by assessing the magnitude of an intervention in patients with symptomatic partial or full thickness tendon tears (Table 2).

There are very-low and low-quality evidences of the VISA-G, for sufficient responsiveness as compared with other PROMs and before and after surgery and rehabilitation with an MIC of 29.0 points (Table 3).

#### VISA-H

Only the development study [4] tested hypotheses for responsiveness by comparing the VISA-H change scores with the Nirschl phase rating scale and a generic tendon grading system or by assessing the magnitude of a conservative intervention in patients with proximal hamstring tendinopathy (Table 2).

There is very-low-quality evidence for sufficient responsiveness of the VISA-H as compared with other outcome measures with no information regarding their measurement properties. There is moderate-quality evidence for sufficient responsiveness following rehabilitation with an MIC of 22.0 points (Table 3).

#### VISA-P

Four studies tested hypotheses for responsiveness by comparing the VISA-P change scores with the Nirchl score [61] and the global rating of change scale [18], or by assessing the magnitude of a surgical or a conservative intervention in patients with patellar tendinopathy (Table 2) [17, 18, 61, 62].

There is high-quality evidence for sufficient responsiveness of the VISA-P as compared with other outcome measures, and low-quality evidence for sufficient responsiveness following physiotherapy with an MIC of 16.0 points (Table 3).

### Interpretability and feasibility

**Fig. 3:**
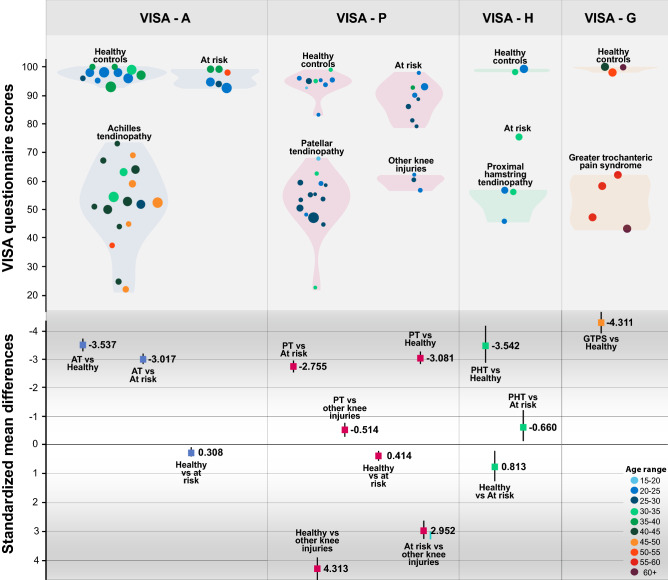
Upper portion shows mean values and normalised distribution (violin) of the VISA scores according to lower limb tendinopathy and groups of individuals included in each study. Lower portion shows the standardized mean differences in group comparisons with effect sizes in standardized mean differences. Data are depicted according to age groups and the size of each circle is proportional to the sample size. In studies reporting median and interquartile range we calculated the mean [36] and standard deviation [63] from relevant equations. For standardized mean difference calculations, we used the pooled weighted values for each comparison

The distribution of the VISA scores and the group differences for patients and other groups of individuals according to each lower limb tendinopathy are depicted in Fig. 3.

One study per VISA calculated the MIC using anchor-based methods. The MIC in 15 patients with insertional Achilles tendinopathy [40] was 6.5, in 56 patients with symptomatic partial or full thickness gluteal tendon tears [13] was 29.0, in 16 patients with proximal hamstring tendinopathy [4] was 22.0, and in 90 patients with patellar tendinopathy [18] was 16.0 points.

Most of the studies did not report on missing items. Three studies reported no missing items [2, 4, 14], while in one study [53] described that 10.6% of the administered questionnaires were incomplete or erroneously filled. No study identified floor and ceiling effects of the scores of patients with tendinopathy; however, a group ceiling effect in studies was seen in asymptomatic individuals [14, 22].

The VISA questionnaires are free to use, self-administered, require no equipment, no specialized training, minimum of communication between administrator and patient, and they are not diagnostic tools. Average completion time for VISA-A and VISA-P was less than 5 min, while for VISA-G ranged from 1.2 to 8.5 min and 2.1 min to 10 min in asymptomatic individuals and patients, respectively. No information was reported for VISA-H completion time.

## Discussion

The most important finding of this study was that the VISA questionnaires presented sufficient reliability, measurement error, construct validity, and responsiveness with variable quality of evidence. Only the VISA-A displayed insufficient measurement error.

There is moderate-quality evidence for sufficient VISA-A, VISA-G, and VISA-P reliability, moderate-quality evidence for sufficient VISA-G and VISA-P measurement error, high-quality evidence for sufficient VISA construct validity, as well as high-quality evidence for sufficient responsiveness only for VISA-A in patients with insertional Achilles tendinopathy following conservative interventions. The evidence for the rest of the measurement properties in VISA questionnaires was sufficient and of low and very-low qualities.

### Test–retest reliability, stability of the condition, and recall bias

An important assumption made in reliability evaluation is that patients are stable on the construct to be measured between the repeated measurements [48]. The selection of an appropriate time interval for test and retest depends on the interplay of two inversely related domains: recall bias and stability of the clinical condition. The time interval should be short enough to ensure that patients are stable and at the same time long enough to prevent recall bias [48]. The quality evaluation of the reliability and measurement error in all included studies was substantially affected (all downgraded for risk of bias) by these two domains. Most studies failed to provide evidence that patients were stable at the second administration of the PROM, or provided evidence of significant differences between test and retest in patients with chronic Achilles tendinopathy [10, 21, 54]. Methods to measure the stability of the condition have been proposed, such as asking the patients to self-rate their condition as unchanged at the second administration of the PROM or using a global rating of change scale [29, 48]. Instead, most studies attempted to ensure stability of the condition by decreasing the time between the repeated administrations and consequently increasing the risk of recall bias. It can be assumed that the symptoms of a chronic lower limb tendinopathy would not change within a week; however, 72% of the included studies did not report the duration of symptoms of the included tendinopathy sample making this assumption unsafe. The possibility of recruitment of patients with ongoing tendinopathy could not be excluded, where a significant improvement or deterioration can be experienced in a short period of time with decreased or continued activity and tendon load [41]. We suggest future studies assessing PROMs’ reliability and measurement error to carefully define an adequate time interval between repeated measurements by avoiding treatment or consultation with a health care provider, asking the patients to confirm that their clinical condition has not changed, ensuring similar conditions in PROMs administration, and following the recommended standards for reporting participant characteristics in tendinopathy research (i.e., symptoms duration) [48, 50].

The pooled or summarized reliability coefficients for the VISA questionnaires displayed sufficient reliability with values greater than 0.82. The pooled ICC estimates presented substantial heterogeneity despite the subgroup analyses; thus, these results should be interpreted with caution. Exploratory inclusion of ICC moderators did not: (a) substantially affect the pooled estimate; (b) decrease the heterogeneity; or (c) suggest moderation by the subgroup of participants.

Although measurement error (SDC) of the VISA questionnaires requires further evaluation; VISA-G, VISA-H, and VISA-P displayed moderate quality of sufficient measurement error not exceeding the MIC. A change in VISA score greater than 4.0, 4.0, and 11.0 points represents a true change for VISA-G, VISA-H, and VISA-P; respectively. The VISA-A only displayed insufficient measurement error; however, larger scale responsiveness studies are required to assess the MIC in other subgroups except insertional Achilles tendinopathy patients. Despite that SDC has significant clinical utility, 53% of the included studies did not report values for measurement error suggesting the need for future studies to evaluate measurement error in patients of different ages and levels of physical activity, or different subgroups of patients within the clinical spectrum of tendinopathy. Moreover, it is suggested that future studies present the differences between test and retest using Bland–Altman methods as this method shows a relationship between the plotted differences and the magnitude of measurements (i.e., proportional error), depicting any systematic bias (i.e., absolute systematic error) and identifies possible outliers allowing meaningful clinical inferences [3].

### Construct validity and hypotheses testing of the VISA questionnaires

The extent to which the results of hypotheses testing for construct validity are consistent with the predefined hypotheses will be evidence supporting validity of the PROM [23]. The VISA questionnaires exhibited high-quality evidence for sufficient known group’s validity, demonstrating that the VISA total score can validly discriminate patients from asymptomatic or at-risk individuals. Pooled weighted VISA scores of patients as compared to asymptomatic and at-risk individuals presented very large effect sizes, in contrast to the significant, but small effect size, differences between groups without tendinopathy (Fig. 3).

Construct validity of a PROM is preferably tested against a “gold standard” [48]. To our knowledge, a gold standard outcome measure does not exist in tendinopathy, as well as for many musculoskeletal conditions which are accompanied with functional disability and pain [23, 45]. Hence, construct validity can be assessed by comparing the PROM of interest with other PROMs that measure a similar construct. In our review, 50% of the included studies used as comparator scales PROMs without information about their reliability and validity, while 32% used SF-36 and 27% region-specific valid and reliable PROMs. Despite that tendinopathy has a unique clinical presentation that significantly differs from other lower limb musculoskeletal conditions [41], region-specific PROMs would be more appropriate for future studies assessing construct validity of the VISA questionnaires (i.e., Lower Extremity Functional Scale, Foot and Ankle Outcome Score), rather than generic or non-validated scales and PROMs.

### Responsiveness and interpretation of the VISA scores

For a PROM to be clinically useful, it must first be psychometrically sound in terms of reliability and validity, but also must be able to detect real change in health status (sensitivity to change) and display the ability to detect absence of change when there is no real change (specificity to change) [7, 8]. From a clinical perspective, the MIC score can be used in establishing a therapeutic threshold in lower limb tendinopathy through the VISA questionnaires. However, beyond inherent methodological limitations in MIC calculation [7, 8], such as the use of distribution or anchor-based methods, or the use of “a little better” or “much better” as the cut-off value from a global rating of change scale, several other factors seem to influence the stability and mediate the variability of MIC score. The potential usefulness of the MIC as a single point estimate for both researchers and clinicians, contrasts with evidence suggesting that the stability of a single MIC score remains an elusive notion in the area of interpretability [6–8].

Moreover, the MIC is context-specific, is not a fixed property of a PROM, and is dependent on characteristics of the population, condition severity, chronicity, intervention, and period of follow-up [7, 57]. To illustrate: a 6.5-point improvement which exceeds the MIC for insertional Achilles tendinopathy following a 12-week conservative intervention has a different meaning for patients with higher levels of disability (i.e., baseline VISA-A score of 38 points—self-rated significant improvement reported by 80% of the patients) [40] compared to lower levels of disability (i.e., baseline VISA-A score of 53 points—self-rated significant improvement by 46% of the patients) [52].

### Strengths and weaknesses of the review, and future study recommendations

Despite the limitations in reliability evaluation, the VISA questionnaires displayed consistently sufficient reliability across studies and groups, suggesting that test–retest reliability should not be a priority when developing new language versions. Rather, resources should be directed towards assessment of other clinimetric properties, such as content and construct validity, measurement error, and responsiveness.

All VISA questionnaires have been categorized as “B” PROMs, meaning that may have the potential to be recommended, but further content and structural validation studies are needed to assess their quality [27]. Clinicians and researchers should interpret the measurement error of the PROMs with caution, given its dependence on MIC, and remain mindful that these scores are patient-population-specific (not generalizable). With regard to responsiveness, future studies should: elucidate how the baseline characteristics can be separated from regression to the mean, standardize methods of assessment, evaluate the MIC scores in subgroups of tendinopathy across the spectrum of the condition, and establish a range of values (instead of a single point estimate) for intervention outcomes.

A degree of subjectivity was necessary in the rating of the standards of the criteria of these newly formed guidelines, though the involvement of three reviewers and the pre-specified criteria helped to minimize the possibility of bias.

The post hoc decision for statistical analyses is acknowledged as a limitation. In addition, given the lack of guidelines performing meta-analyses using the ICC, the robustness of the assumptions we made for estimating the group effect remains to be investigated.

Finally, the exclusion of studies that only used a VISA questionnaire as an outcome measurement instrument (i.e., randomized controlled trials) following COSMIN suggestions can be considered as a limitation. It can be suggested to the COSMIN developers to consider this especially with regard to the clinimetric domains of construct validity and responsiveness in future guideline updates.

## Conclusion

The VISA questionnaires seem to have sufficient clinimetric evidence for reliability, measurement error, construct validity, and responsiveness, except VISA-A that displayed insufficient clinimetric evidence for measurement error. Lack of adherence to guidelines significantly affected the quality of evidence for VISA reliability and measurement error. In construct validity (convergent) evaluation, the majority of the comparator instruments were non condition specific or lacked sufficient psychometric properties. Updating and modifications of the VISAs are required to reflect the needs across the spectrum of age, activity, and functional capacity of patients with lower limb tendinopathies.

## Supplementary Information

Below is the link to the electronic supplementary material.

Supplementary file1 (DOCX 16 kb)

Supplementary file2 (XLSX 20 kb)

## Abbreviations

CI: Confidence interval
COSMIN: Consensus-based Standards for the selection of health Measurement Instruments
ICC: Intraclass correlation coefficient
MIC: Minimally important change
PROM: Patient-reported outcome measures
PRISMA: Preferred Reporting Items for Systematic Reviews and Meta-Analyses
SF36: Medical Outcomes Study 36-Item Short-Form Health Survey
SDC: Smallest detectable change
SEM: Standard error of measurement
SMD: Standardized mean differences
VISA: Victorian Institute of Sport Assessment
VISA-A: Victorian Institute of Sport Assessment Achilles tendinopathy
VISA-G: Victorian Institute of Sport Assessment greater trochanteric pain syndrome
VISA-H: Victorian Institute of Sport Assessment proximal hamstring tendinopathy
VISA-P: Victorian Institute of Sport Assessment patellar tendinopathy
