# Supplementary material for: A systematic review evaluating the clinimetric properties of the Victorian Institute of Sport Assessment (VISA) questionnaires for lower limb tendinopathy shows moderate to high-quality evidence for sufficient reliability, validity and responsiveness—part II
Source: Knee Surg Sports Traumatol Arthrosc. 2021 Apr 16;29(9):2765–88. doi: 10.1007/s00167-021-06557-0 (PMC8384816; doi:10.1007/s00167-021-06557-0)
Supplement: Supplementary file 1 — Supplementary file1 (DOCX 16 kb) [file 167_2021_6557_MOESM1_ESM.docx]

**Online Resource 1**

**Evaluating lower limb tendinopathy with Victorian Institute of Sport Assessment (VISA) questionnaires. Part II: a systematic review of the clinimetric evidence of reliability, validity, and responsiveness according to COSMIN methodology**

**Knee Surgery, Sports Traumatology, Arthroscopy**

Vasileios Korakakis^1,2^

Rod Whiteley^1^

Argyro Kotsifaki^1^

Manos Stefanakis^3^

Yiannis Sotiralis^2^

Kristian Thorborg^4^

^1^ Aspetar Orthopaedic and Sports Medicine Hospital, Doha, Qatar.

^2^ Hellenic Orthopaedic Manipulative Therapy Diploma (HOMTD), Athens, Greece.

^3^ University of Nicosia, School of Science, Program of Physiotherapy, Nicosia, Cyprus.

^4^ Department of Orthopaedic Surgery, Sports Orthopedic Research Center – Copenhagen (SORC-C), Amager-Hvidovre Hospital, Faculty of Health Sciences, Copenhagen University, Copenhagen, Denmark.

Address correspondence to Vasileios Korakakis

Aspetar, Orthopaedic and Sports Medicine Hospital, PO 29222, Doha, Qatar.

E-mail: Vasileios.Korakakis@aspetar.com

**Search strategy (for PUBMED)**

(((((((((visa[Title/Abstract]) OR visa-a[Title/Abstract]) OR visa-p[Title/Abstract]) OR visa-g[Title/Abstract]) OR visa-h[Title/Abstract]) OR Victorian Institute of Sport Assessment[Title/Abstract])) AND ((((((((((patella* tend*[Title/Abstract]) OR patella* tendin*[Title/Abstract]) OR jumper’s knee[Title/Abstract]) OR achilles tendinopathy[Title/Abstract]) OR achilles tendin*[Title/Abstract]) OR gluteal tendinopathy[Title/Abstract]) OR gluteal tendin*[Title/Abstract]) OR trochanteric pain syndrome[Title/Abstract]) OR hamstring* tendin*[Title/Abstract]) OR trochanteric tendin*[Title/Abstract])) AND ((instrumentation[sh] OR methods[sh] OR "Validation Studies"[pt] OR "Comparative Study"[pt] OR "psychometrics"[MeSH] OR psychometr*[tiab] OR clinimetr*[tw] OR clinometr*[tw] OR "outcome assessment (health care)"[MeSH] OR "outcome assessment"[tiab] OR "outcome measure*"[tw] OR "observer variation"[MeSH] OR "observer variation"[tiab] OR "Health Status Indicators"[Mesh] OR "reproducibility of results"[MeSH] OR reproducib*[tiab] OR "discriminant analysis"[MeSH] OR reliab*[tiab] OR unreliab*[tiab] OR valid*[tiab] OR "coefficient of variation"[tiab] OR coefficient[tiab] OR homogeneity[tiab] OR homogeneous[tiab] OR "internal consistency"[tiab] OR (cronbach*[tiab] AND (alpha[tiab] OR alphas[tiab])) OR (item[tiab] AND (correlation*[tiab] OR selection*[tiab] OR reduction*[tiab])) OR agreement[tw] OR precision[tw] OR imprecision[tw] OR "precise values"[tw] OR test-retest[tiab] OR (test[tiab] AND retest[tiab]) OR (reliab*[tiab] AND (test[tiab] OR retest[tiab])) OR stability[tiab] OR interrater[tiab] OR inter-rater[tiab] OR intrarater[tiab] OR intra-rater[tiab] OR intertester[tiab] OR inter-tester[tiab] OR intratester[tiab] OR intra-tester[tiab] OR interobserver[tiab] OR inter-observer[tiab] OR intraobserver[tiab] OR intra-observer[tiab] OR intertechnician[tiab] OR inter-technician[tiab] OR intratechnician[tiab] OR intra-technician[tiab] OR interexaminer[tiab] OR inter-examiner[tiab] OR intraexaminer[tiab] OR intra-examiner[tiab] OR interassay[tiab] OR inter-assay[tiab] OR intraassay[tiab] OR intra-assay[tiab] OR interindividual[tiab] OR inter-individual[tiab] OR intraindividual[tiab] OR intra-individual[tiab] OR interparticipant[tiab] OR inter-participant[tiab] OR intraparticipant[tiab] OR intra-participant[tiab] OR kappa[tiab] OR kappa's[tiab] OR kappas[tiab] OR repeatab*[tw] OR ((replicab*[tw] OR repeated[tw]) AND (measure[tw] OR measures[tw] OR findings[tw] OR result[tw] OR results[tw] OR test[tw] OR tests[tw])) OR generaliza*[tiab] OR generalisa*[tiab] OR concordance[tiab] OR (intraclass[tiab] AND correlation*[tiab]) OR discriminative[tiab] OR "known group"[tiab] OR "factor analysis"[tiab] OR "factor analyses"[tiab] OR "factor structure"[tiab] OR "factor structures"[tiab] OR dimension*[tiab] OR subscale*[tiab] OR (multitrait[tiab] AND scaling[tiab] AND (analysis[tiab] OR analyses[tiab])) OR "item discriminant"[tiab] OR "interscale correlation*"[tiab] OR error[tiab] OR errors[tiab] OR "individual variability"[tiab] OR "interval variability"[tiab] OR "rate variability"[tiab] OR (variability[tiab] AND (analysis[tiab] OR values[tiab])) OR (uncertainty[tiab] AND (measurement[tiab] OR measuring[tiab])) OR "standard error of measurement"[tiab] OR sensitiv*[tiab] OR responsive*[tiab] OR (limit[tiab] AND detection[tiab]) OR "minimal detectable concentration"[tiab] OR interpretab*[tiab] OR ((minimal[tiab] OR minimally[tiab] OR clinical[tiab] OR clinically[tiab]) AND (important[tiab] OR significant[tiab] OR detectable[tiab]) AND (change[tiab] OR difference[tiab])) OR (small*[tiab] AND (real[tiab] OR detectable[tiab]) AND (change[tiab] OR difference[tiab])) OR "meaningful change"[tiab] OR "ceiling effect"[tiab] OR "floor effect"[tiab] OR "Item response model"[tiab] OR IRT[tiab] OR Rasch[tiab] OR "Differential item functioning"[tiab] OR DIF[tiab] OR "computer adaptive testing"[tiab] OR "item bank"[tiab] OR "cross-cultural equivalence"[tiab]))) NOT ((‘delphi-technique’[ti] OR cross-sectional[ti] OR "addresses"[Publication Type] OR "biography"[Publication Type] OR "case reports"[Publication Type] OR "comment"[Publication Type] OR "directory"[Publication Type] OR "editorial"[Publication Type] OR "festschrift"[Publication Type] OR "interview"[Publication Type] OR "lectures"[Publication Type] OR "legal cases"[Publication Type] OR "legislation"[Publication Type] OR "letter"[Publication Type] OR "news"[Publication Type] OR "newspaper article"[Publication Type] OR "patient education handout"[Publication Type] OR "popular works"[Publication Type] OR "congresses"[Publication Type] OR "consensus development conference"[Publication Type] OR "consensus development conference, nih"[Publication Type] OR "practice guideline"[Publication Type]) NOT ("animals"[MeSH Terms] NOT "humans"[MeSH Terms]))
